# K-mer based prediction of *Clostridioides difficile* relatedness and ribotypes

**DOI:** 10.1099/mgen.0.000804

**Published:** 2022-04-06

**Authors:** Matthew Phillip Moore, Mark H. Wilcox, A. Sarah Walker, David W. Eyre

**Affiliations:** ^1^​ Big Data Institute, Nuffield Department of Population Health, University of Oxford, Oxford, UK; ^2^​ Nuffield Department of Medicine, University of Oxford, Oxford, UK; ^3^​ NIHR Oxford Biomedical Research Centre, University of Oxford, Oxford, UK; ^4^​ Healthcare Associated Infection Research Group, Leeds Teaching Hospitals NHS Trust and University of Leeds, Leeds, UK; ^5^​ NIHR Health Protection Research Unit in Healthcare Associated Infections and Antimicrobial Resistance at University of Oxford in partnership with Public Health England, Oxford, UK

**Keywords:** *Clostridioides difficile*, k-mer, MASH, ribotype

## Abstract

Comparative analysis of *Clostridioides difficile* whole-genome sequencing (WGS) data enables fine scaled investigation of transmission and is increasingly becoming part of routine surveillance. However, these analyses are constrained by the computational requirements of the large volumes of data involved. By decomposing WGS reads or assemblies into k-mers and using the dimensionality reduction technique MinHash, it is possible to rapidly approximate genomic distances without alignment. Here we assessed the performance of MinHash, as implemented by sourmash, in predicting single nucleotide differences between genomes (SNPs) and *C. difficile* ribotypes (RTs). For a set of 1905 diverse *C. difficile* genomes (differing by 0–168 519 SNPs), using sourmash to screen for closely related genomes, at a sensitivity of 100 % for pairs ≤10 SNPs, sourmash reduced the number of pairs from 1 813 560 overall to 161 934, i.e. by 91 %, with a positive predictive value of 32 % to correctly identify pairs ≤10 SNPs (maximum SNP distance 4144). At a sensitivity of 95 %, pairs were reduced by 94 % to 108 266 and PPV increased to 45 % (maximum SNP distance 1009). Increasing the MinHash sketch size above 2000 produced minimal performance improvement. We also explored a MinHash similarity-based ribotype prediction method. Genomes with known ribotypes (*n*=3937) were split into a training set (2937) and test set (1000) randomly. The training set was used to construct a sourmash index against which genomes from the test set were compared. If the closest five genomes in the index had the same ribotype this was taken to predict the searched genome’s ribotype. Using our MinHash ribotype index, predicted ribotypes were correct in 780/1000 (78 %) genomes, incorrect in 20 (2 %), and indeterminant in 200 (20 %). Relaxing the classifier to 4/5 closest matches with the same RT improved the correct predictions to 87 %. Using MinHash it is possible to subsample *C. difficile* genome k-mer hashes and use them to approximate small genomic differences within minutes, significantly reducing the search space for further analysis.

## Data Summary

**Impact Statement**

The genetic code, or DNA, of bacteria is increasingly used to track how infection spreads and to guide infection control interventions, as similar or identical DNA sequences are expected in samples from pairs of individuals related by transmission. While obtaining the DNA sequence for bacteria is increasingly straightforward, comparing thousands or even millions of sequences requires substantial computing power and time using current approaches. Here we describe how a method for summarising sequencing data, MinHash, can be used to rapidly reduce the number of possible close sequence matches in *Clostridioides difficile *, an important healthcare-associated pathogen. It can also be used to approximate traditional schemes used to classify *C. difficile* into smaller subgroups in transmission analyses, such as ribotyping.

The authors confirm all supporting data, code and protocols have been provided within the article or through supplementary data files.

## Introduction

Whole genome sequencing (WGS) has transformed our understanding of the epidemiology of many bacterial pathogens. For example, *Clostridioides* (formerly *Clostridium *) *difficile*, previously believed to be transmitted predominantly from other hospitalised cases, has been shown to be mostly acquired from other sources [1]. WGS also facilitates outbreak investigations, e.g. of enteric pathogens [2–4] and highly drug-resistant gonorrhoea [5, 6], and can support specific infection control interventions [7] and surveillance [8]. However, all these potential applications rely on being able to identify closely genetically related infections against an ever-growing collection of previously sequenced genomes. To date most studies have relied on constructing phylogenies on the basis of single nucleotide polymorphisms (SNPs), however such approaches are computationally demanding, and increasingly impractical for large scale comparisons of genomes for timely public health or infection control interventions.

One potential approach to this challenge is to ‘divide and conquer’, such that a rapid screen is used to define which subgroup of a species a genome of interest is from, reducing the number of genomes that need to be considered in a more detailed analysis. For example, multi-locus sequencing typing (MLST), based in *C. difficile* on seven housekeeping genes [9], can be used to rapidly type isolates from sequencing reads without the need for mapping or assembly [10]. MLST can eliminate the possibility of an outbreak if isolate sequences have diverse sequence types (STs) but lacks the discriminatory precision to identify whether sequences of the same ST constitute a likely outbreak. Core genome MLST (cgMLST) expands on MLST providing, in theory, a standardised set of 2000–3000 *C *. *difficile* core genes [11] for far greater differentiation of isolates by genomic data. The selection of cgMLST loci for schema creation, quality assessment and designation of profiles, and ongoing curation of cgMLST schemes, have been made possible by open-source software [12]. However, there are multiple cgMLST schemes for *C. difficile* that are not readily inter-operable with open-source software [12] due to differences in reference genome annotation and gene selection protocols. To address these limitations and improve cgMLST for outbreak detection, a hash based cgMLST scheme has been developed for *C. difficile,* removing the need for a centralised, curated database [13]. Other typing schemes can also be used to subdivide isolates. For *C. difficile *, PCR ribotyping [14] remains the standard for non-WGS-based surveillance. However, identifying PCR ribotypes directly from WGS based on short reads is challenging. An *in silico* ribotyping method has been developed to detect ribotype (RT) 027, but not ribotypes more generally [15]. There is not a perfect 1 : 1 correspondence between ribotypes and STs, but common equivalents, e.g. ST1/RT027 are well described [16].

We therefore investigated the performance of alternative approaches based on k-mers for screening isolates to identify subsets with closely related genome sequences. K-mers are fragments of sequence data of length k. Therefore, these approaches are potentially species agnostic, and could be deployed widely, without the need for species-specific schemes such as MLST, cgMLST or ribotyping. They also have the advantage that they do not necessarily require prior genome assembly or alignment as required for cgMLST or SNP based analyses respectively. Bacterial genome sequences contain millions of k-mers and so dimensionality reduction is used to improve performance. The MinHash dimensionality reduction method is a form of local sensitivity hashing [17] that was first applied to comparative genomics with the development of MinHash extension, Mash [18]. With Mash, and similar k-mer-based dimensionality reduction techniques developed subsequently [19–21], it is now possible to quickly compare tens of thousands of genomes and rapidly identify organisms in metagenomic sequencing projects without alignment [22]. In one example 54118 organisms, comprising 618 Gbp of sequencing data, resulted in Mash sketches totalling 93 MB, a 7000-fold compression compared with uncompressed fasta. Sketching and ~1.5 billion pairwise sketch comparisons took 40 CPU hours for a k-mer size of 21 and sketch size of 500, increasing to 115.2 CPU hours for a sketch size of 5000 [18]. These methods depend on a genome k-mer ‘sketch’ (a random subset of all k-mers of a certain size) and as such precision, in theory, may be increased by increasing the sketch size with the cost of increased compute time. The precision of these methods also depends upon biological factors such as how correlated core genome SNP distances are with accessory gene differences within a species or lineage of interest [23].

Here we test the performance of k-mer based algorithms in *C. difficile* and assess whether these algorithms can be used to approximate the most common typing scheme in use, ribotyping, and identify sequence pairs within SNP thresholds in order to provide backwards compatibility with existing surveillance systems and to identify groups of closely related isolates for in depth phylogenetic characterisation.

## Methods

### Data

Previously sequenced genomes from studies of clinical isolates in the CD-link study (six UK hospitals) (*n*=973), Leeds (*n*=1876), *C. difficile* UK ribotyping network (*n*=684), EUCLID and CLOSER studies (*n*=678), Leeds and ECDC collections (*n*=369), RT078 genomes from the NCBI short read archive (*n*=68) and RT244 from Australia (*n*=16) with available ribotypes were considered for inclusion (*n*=4655). The large majority of ribotypes in the study were assigned by the national *C. difficile* reference laboratory in Leeds, UK using capillary gel electrophoresis as previously described [24]. Details of included sequences, short-read archive accession numbers and ribotypes are provided in Table S1.

### Bioinformatic processing

K-mer based comparisons were undertaken with and without prior genome assembly. Use of assemblies potentially allows most sequencing errors to be removed first, but is more computationally intensive. Sequencing adapters were removed using BBDuk [25]. Reads were assembled using Velvet [26] with Velvet optimiser (https://github.com/tseemann/VelvetOptimiser), without prior read quality trimming or filtering. Contigs <1000 bp in length were filtered out of assemblies to avoid fragmented assemblies. Assembly quality was assessed using QUAST-5.0.2 [27]. Assemblies <3.7 Mb or >4.7 Mbp total assembly size were excluded to avoid genomes with insufficient data or potential contamination (*n*=163 and *n*=53 respectively). Assemblies with >1000 contigs were also excluded to avoid excessively fragmented assemblies (*n*=109). Multi-locus sequence types (STs) were determined *in silico* from assemblies using mlst [28] and the scheme of Griffiths *et al*. [9]. Those without a determinable *C. difficile* MLST were excluded (*n*=297). We screened the dataset for potential sample mislabelling by looking for incongruent ST-ribotype pairs with a novel ST-RT network approach. The Python package NetworkX [29] was used to generate a weighted, undirected network with each ST and RT as a node in the network. Each genome sharing an ST and RT added an edge and an edge weight of one between the ST and RT nodes. Potentially mislabelled samples were screened for as edges with low weights where there was strong evidence for an alternative ST-RT relationship: for all connected components with >3 nodes, an edge (connecting node A and B) was considered potentially spurious if the edge weight was less than the sum of edge weights connected to node A divided by ten and also less than the sum of edge weights connected to node B divided by ten. Samples were excluded on this basis (*n*=96), as re-running the sequencing and ribotyping for this current study was not practical. A random sample of 1905 genomes were taken to assess k-mer vs SNP distances. For analyses without prior genome assembly reads were quality trimmed using TrimGalore! [30, 31] with minimum Phred Score 30 and low abundance k-mers removed using khmer trim low abundance [32, 33] requiring ten or more copies of each k-mer.

### Comparison of K-mer based distances with SNP distances

We assessed the performance of several k-mer based approaches. Sourmash [21] was used to generate MinHash Jaccard distances.

To explore explanations for discrepancies between k-mer based metrics and SNPs, in addition to the actual sequencing data described above, we simulated perfect Illumina short sequence reads and reads with sequencing errors using wgsim [34]. Three sets of 500 *C *. *difficile* simulated genome read datasets were simulated using the reference genome 630 [35] (length=4 290 252 bp) with a mutation rate set randomly to achieve an expected number of SNPs of between 1 and 500 from the reference genome. Coverage depth was set to 70 x. The first simulations comprised SNPs only, the second SNPs with 15 % of polymorphisms as indels and the third SNPs with a sequencing error rate of 0.01. In simulations with indels, the default probability that an indel is extended (0.30) was applied. Simulated assemblies were generated by addition of simulated variants to the CD630 genome sequence from wgsim output for each genome. Real pairwise SNP and indel differences between simulated reads and assemblies were recorded from wgsim output.

We analysed the capacity of the k-mer based algorithms to identify sequence pairs within SNP thresholds such as ≤10 SNPs, i.e. samples sufficiently closely related to be related by transmission within the last 5 years [1]. Results were also generated for ≤50 SNPs and ≤100 SNPs for comparison. For varying thresholds in the sourmash distance (denoted d_kmer_), we evaluated the number of true positive pairs (TP), i.e. ≤10 SNPs and distance ≥d_kmer_ (d_kmer_=1 denotes smallest sourmash distance possible and d_kmer_ <1 increasingly dissimilar to d_kmer_=0) and false positive pairs (FP), i.e. >10 SNPs and distance ≥d_kmer_. Sensitivity, specificity and positive predictive value (PPV) were calculated. The PPV value represents the proportion of genome pairs ≥d_kmer_ that are true positives. The reduction in search space was calculated as the proportion of overall genome pairs <d_kmer_. NetworkX [29] was used to cluster genomes by sourmash distance. Full k-mer hash set comparisons were performed with Dashing [19] khash for comparison (Table S2, Fig. S1). A large sourmash k-mer size was selected (k=51) for sensitivity and the optimal sketch size estimated as the smallest sketch size (s=2000) approximating the performance of full k-mer hash sets (Figs S2 and S3).

Unique k-mers in a genome can arise from SNPs and indels, but also from other genetic material (e.g. accessory genes, contamination or insertions). We evaluated the size of the association between greater than expected k-mer distances versus SNPs and the accessory genes present. Accessory genes were predicted from assemblies using roary [36] with the following settings: core genes were defined as those gene clusters present in ≥99 % isolates; the blast percent identity to designate genes as the same was set to ≥95 % and was repeated at 75 and 50% blast identity. We also evaluated SNP and k-mer distances between sequence data generated from repeated sequencing of the same DNA or from repeated sequencing of the same isolate (Table S1).

### Ribotype prediction

We assessed if k-mer based distances could be used to predict ribotypes directly from sequence reads. Genome sketch signatures were generated with sourmash [21] compute (K=51, sketch size=2000) for all sequenced genomes. An index (sbt) of all genome sketches was generated with sourmash index and searched with sourmash search, with default parameters. Genome assemblies that passed quality filtering and that had reliable ribotypes (*n*=3937) were randomly divided into training (*n*=2,937) and test datasets (*n*=1000). The training dataset was used to generate a sourmash index. We then compared each sequence in the test dataset to all in the training dataset (sourmash index). The RT of the five most closely related genomes (using sourmash search) in the training dataset were identified, considering the accuracy of sourmash search determined ribotypes based on requiring all five closest matches to be the same ribotype and requiring only 4/5 matches to be the same.

## Results

### k-mer distances versus SNPs in simulated data

Using 500 simulated genomes without sequencing errors or indels (124750 pairs, 2–11 SNPs) we evaluated the ability of k-mer distances to identify the 24 (0.02 %) of pairs with ≤10 SNPs. Comparing sourmash signatures from assemblies, at the largest mash distance (0.999) with 100 % sensitivity to identify all 24 ≤10 SNP pairs, the search space was reduced by 98.5 % (to 1841 from 124 750 genome pairs), with a PPV of 1.3 % (24/1841) (Table S4, Fig. S4). As expected (assuming no SNPs are within k base pairs of one another), the full jaccard distance from all k-mers (dashing khash) predicted ≤10 SNP pairs with 100 % sensitivity and a PPV of 100 %. In simulated genomes with SNPs and indels, and mash distance threshold 0.999, the search space reduction was 98.4 % (1970/124750), with a PPV of 1.9 % (37/1970) (Table S4, Fig. S4). Using full k-mer jaccard distance for this simulation at 100 % sensitivity predicted ≤10 SNP pairs with a PPV of 56.1 % (37/66). Performance for the SNPs only and SNPs and indels simulations were very similar when sourmash signatures were generated directly from sequencing reads (Table S4, Fig. S4). A third set introducing high sequencing error (at a rate of 0.01, with x70 coverage) markedly reduced performance from reads by comparison (Table S4, Fig. S4). The simulation had only SNPs and a high level of sequencing errors, and genome pairs differed by three to 1046 SNPs (24 pairs ≤10 SNPs) (Table S4, Fig. S4). Performance at 100 % sensitivity (mash distance threshold 0.105), produced only a 10 % reduction in search space (111502/124 750 genome pairs) with a PPV of 0.02 % (24/111 502).

### k-mer distances versus SNPs in real data

A random subsample of *C. difficile* genomes (*n*=1905) was selected to assess the precision of mash distances at predicting small SNP cut-offs in real data. The genomes ranged from 0 to 168 519 SNPs different from one another; of 1 813 560 pairs, 52 020 (2.9 %) were within ≤10 SNPs, in 172 clusters, each containing a median (range) 3 (1–39 716) pairs.

**Fig. 1.**
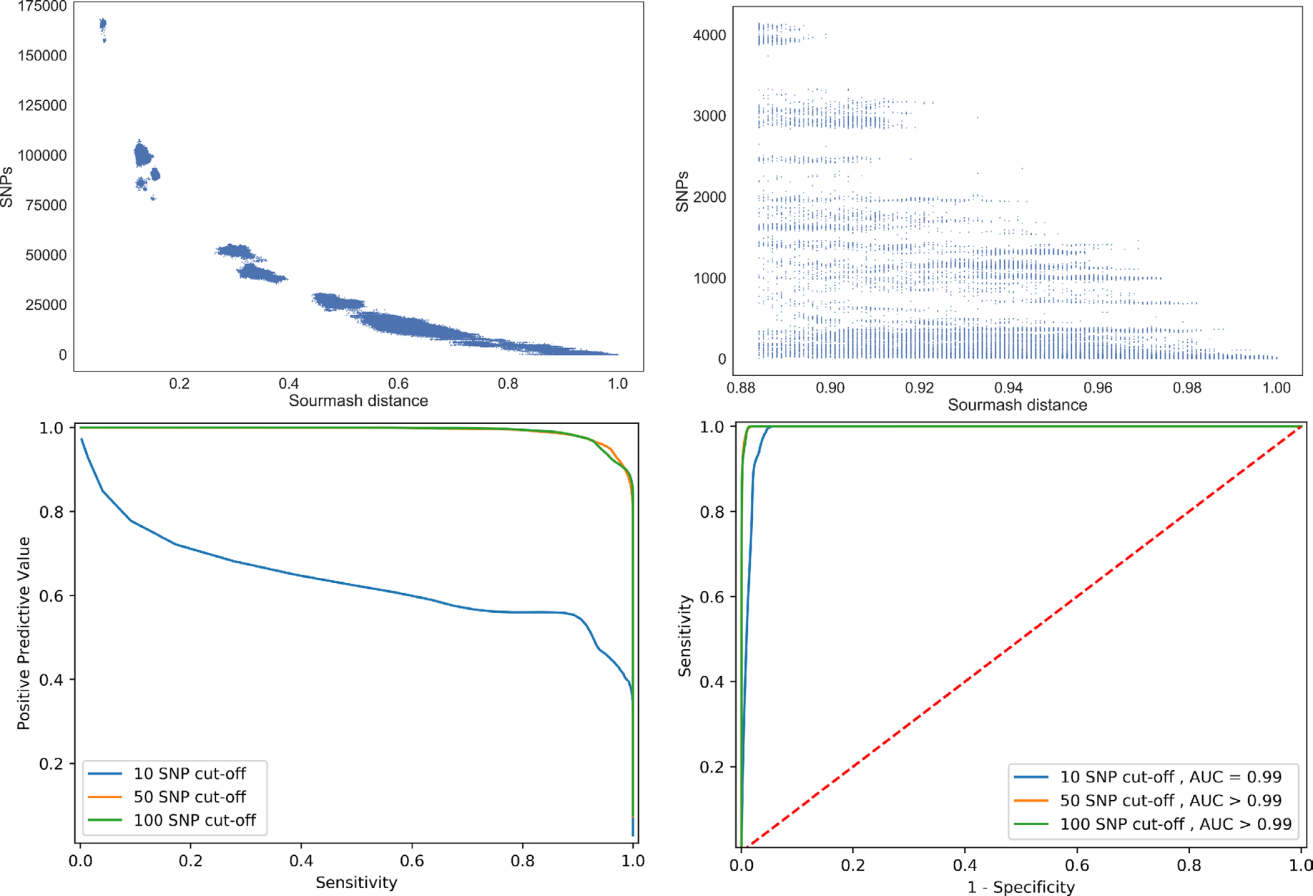
The relationship between and measures of performance of non-redundant pairwise sourmash distances from assembled genomes and their corresponding core genome SNP distances. Top left shows a scatterplot of sourmash distances vs SNP distances (*n*=1 813 560) comparisons and top right, those ≥0.884 sourmash distance where sensitivity for pairs≤10 SNPs is 100 %. Bottom left shows performance for all sourmash distance thresholds predicting pairs that are ≤100 SNPs, ≤50 SNPs and ≤10 SNPs by positive predictive value vs sensitivity and bottom right the receiver operator curve with area under the curve values rounded to two decimal places.

Using sourmash signatures from assembled genomes, the largest mash distance (0.884) with 100 % sensitivity for identifying all≤10 SNP pairs, reduced the search space by 93.9 % (to 109 914/1 813 560 genome pairs) with a PPV of 32.1 % (52 020/161 934) (Fig. 1, Tables 1 and S3). Pairs≥0.884 sourmash distance apart fell into 49 clusters containing a median (range) 28 (1 - 99681) pairs. The largest SNP difference within a sourmash-identified cluster was 4144. At the largest sourmash distance (0.973) with ≥95 % sensitivity to identify ≤10 SNP pairs, the search space was reduced by 94.0 % (108 266/1 813 560) with a PPV of 45.8 % (49 538/108 266). Pairs≥0.973 sourmash distance apart fell into 119 clusters containing a median (range) 3 (1–88 708) pairs, the largest pairwise SNP difference within a cluster was 1009 (Table 1). Signatures were also generated directly from sequencing reads. This resulted in poorer performance compared with sourmash signatures from assembled genomes (Table 1). Performance was improved by removal of low abundance k-mers (Tables 1 and S5, Fig. S5).

**Table 1.** Performance of k-mer based genome comparisons identifying pairs with ≤10 SNPs. Contigs<1000 bp were removed from assembled genomes before k-mer hash signatures were generated. Results for k-mer hash signatures are presented from sequencing reads with and without removal of low abundance reads. Core gene k-mer hash signatures were generated from multi-fasta files such that overlapping regions did not generate k-mers

K-mer source	Sensitivity for including pairs≤10 SNPs used to determine Sourmash threshold	Sourmash distance threshold	Pairs reduced to (% reduction from 1813560)	Positive Predictive Value (true positives / true positives and false positives)	Clusters identified	Median (range) pairs per cluster	Largest SNP difference within cluster
**Assemblies**	100 % sensitivity for all ≤10 SNPs	0.884	161 934 (↓91.1%)	32.1% (52,020/161,934)	49	28 (1-99,681)	4144
**Assemblies**	95 % sensitivity for all ≤10 SNPs	0.973	108 266 (↓94.0%)	45.8 % (49,538/108,266)	119	3 (1-88,708)	1009
**Reads exc low abundance k-mers**	100 % sensitivity for all ≤10 SNPs	0.780	177 940 (↓90.2%)	29.2% (52,020/177,940)	38	102 (1-99,681)	7705
**Reads exc low abundance k-mers**	95 % sensitivity for all ≤10 SNPs	0.950	125 761 (↓93.1%)	39.5 % (49,528/125,761)	72	5 (1-96,520)	1460
**Reads (all k-mers**)	100 % sensitivity for all ≤10 SNPs	0.071	1 492 029 (↓17.7%)	3.5% (52,020/1,492,029)	1	1 492 029	105 373
**Core genes**	100 % sensitivity for all ≤10 SNPs	0.978	157 114 (↓91.3%)	33.1% (52,020/157,114)	52	40.5 (1-99,681)	3202

**Fig. 2.**
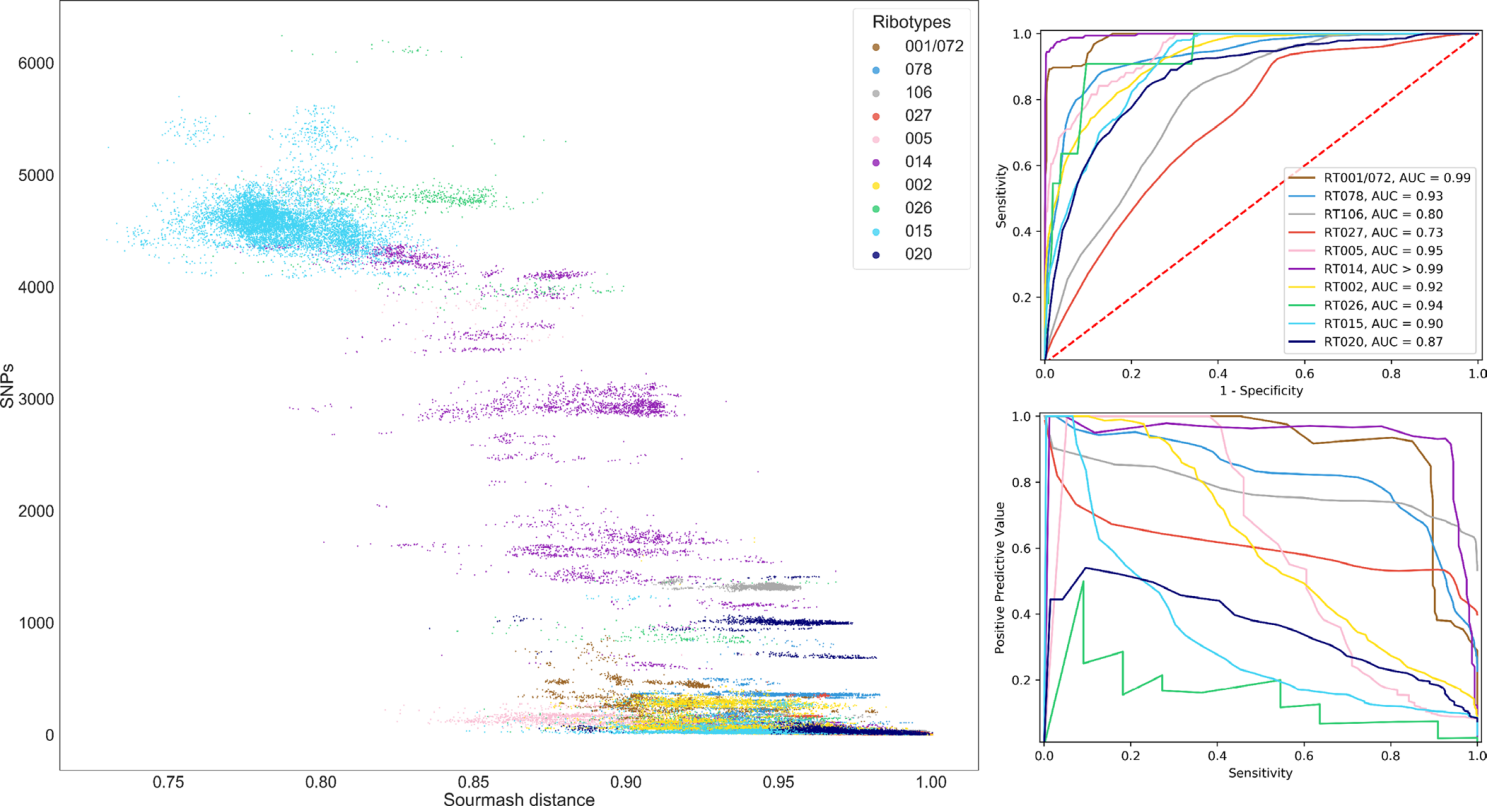
Sourmash and SNP distances within lineage (ribotypes) from assembled genomes for which there were ≥50 genomes per ribotype. Left, a scatterplot shows the relationship between sourmash distance and SNPs within ribotype. Performance of all sourmash thresholds predicting pairs≤10 SNPs within ribotype is plotted top right with a receiver operator curve and bottom right positive predictive values vs sensitivity. Plots are consistently coloured by ribotype and area under the curve values are rounded to two decimal places.

To assess whether k-mer based approaches could improve on existing typing schemes we examined the within-lineage performance of sourmash distance thresholds at 100 % sensitivity for predicting ≤10 SNPs between assembled genomes. This evaluates performance in sets of more similar genome pairs with within-lineage population structure (Fig. 2, Table S6). Ribotypes with ≥50 genomes in the sample were included (005, 015, 027, 014, 106, 020, 002, 078, 026 and 001/072; Table 2). For comparison, we considered chance of a random pair of genomes being within ≤10 SNPs, the random PPV and estimated the ratio of the PPV achieved by the sourmash threshold vs the random PPV. For the whole dataset (not conditioning on ribotype) the ratio of sourmash:random PPV using a distance with 100 % sensitivity to identify pairs with ≤10 SNPs was 11.1 (32.1/2.9). Within ribotypes, the lowest ratio was that of RT078 at 0.8 (24.7/29.4) and the highest RT001/072 at 4.8 (28.9/6) followed by RT014 at 4.1 (12/2.9). In total, in 6/10 ribotypes the sourmash threshold at the 100 % sensitivity distance threshold outperformed the random classifier, though none by as much as the whole dataset (Table 2).

**Table 2.** Performance of k-mer based genome comparisons identifying pairs with ≤10 SNPs. All k-mer hash signatures were generated from assembled genomes per ribotype. Random positive predictive value is the performance of Sourmash threshold 0.000, or proportion of the genome pairs with ≤10 SNPs in the dataset

RT	Median SNPs within lineage	SNP range	Sourmash threshold	Search space reduction	PPV	Random PPV	PPV/Random PPV
**78**	17	0–919	0.932	6,896/7,503 (↓ 8.1%)	25 .%	29.4%	0.8
								**5**	103	0–5074	0.935	940/2,926 (↓67.9%)	8.1%	2.6%	3.1
**27**	13	0–470	0.945	99,564/99,681 (↓0.11%)	40.0%	39.9%	1								
								**26**	943	0–6242	0.924	464/1326 (↓65.0%)	2.4%	2.4%	1
**2**	65	0–1756	0.920	5991/8001 (↓29.4%)	9.5%	6.7%	1.4								
								**20**	44	0–1416	0.947	3422/3828 (↓10.6%)	8.2%	7.4%	1.1
**15**	4428	0–5700	0.884	5061/13 366 (↓62.1%)	8.4%	3.2%	2.6								
								**14**	1756	0–4391	0.939	1353/5671 (↓76.1%)	12.0%	2.9%	4.1
**106**	9	0–1400	0.940	14 454/14 878 (↓2.8%)	55.0%	53.2%	1								
								**001/072**	217.5	0–885	0.959	740/3570 (↓79.3%)	29.00%	6.0%	4.8
**All genomes**	24 576	0–168 519	0.884	161 934/1 813 560 (↓91.1%)	32.1 %	2.9 %	11.1								

One possibility for k-mer based approaches would be to rapidly exclude genome pairs with large SNP distances resulting in clusters of pairs with comparatively smaller distances for further, fine scaled investigations. This process is comparable to clustering by MLST (also rapidly discernible from genome sequences). We compared the SNP distance ranges in sourmash clusters to MLST clusters. The largest SNP distance for instance in sourmash clusters from assembled genomes using a distance with 100 % sensitivity to identify pairs with ≤10 SNPs was 4144 (Table 1). Clustering genomes sharing the same ST, for those with ≥50 genomes per ST (*n*=11), four of the 11 MLST clusters contained larger SNP distances than the largest within sourmash clusters; the largest in ST3 (13 874) and ST7 (10 296). The remaining seven ST clusters contained smaller SNP distances than the largest SNP distance within sourmash clusters.

### Impact of gene differences between assemblies on comparisons

We assessed the number of gene differences between genome pairs using roary to assess whether core genome heterogeneity or accessory gene differences were linked to the performance of the sourmash distance approximation. Correlation between sourmash distance dissimilarity was higher with number of SNPs (Spearman’s rho=0.95, *P*<0.001) than pairwise gene number difference at 95 % blast identity (Spearman’s rho=0.79, *P*<0.001). In pairs within ≤10 SNPs, there was a weaker correlation between sourmash distance dissimilarity and increasing SNPs as expected (Spearman’s rho=0.25, *P*<0.001) but the correlation between sourmash distance dissimilarity and pairwise gene number difference was higher (Spearman’s rho=0.84, *P*<0.001). Trends were concordant at 75 and 50% blast identity (Fig. S6).

We therefore assessed the performance of k-mer distances using extracted core genome alone. Core genes (gene clusters present in ≥99 % isolates) were extracted, signatures were generated based on multi-fasta core gene files and the performance predicting core SNPs assessed. At the largest mash distance (0.978) with 100 % sensitivity to identify all ≤10 SNP pairs, the search space was reduced by 91.3 % (157 114/1 813 560 genome pairs) with a PPV of 33.1 % (52 020/157 114), providing comparable performance with whole genome assemblies overall (PPV 32.1 % at 100 % sensitivity) (Table 1 and Fig. S7, Fig. S7). The correlation between sourmash distance dissimilarity and number of SNPs was also slightly greater (Spearman’s rho=0.97, *P*<0.001) compared with k-mers generated from assemblies.

We also investigated the number of gene differences reported by roary between replicate sequences. Following quality filtering, 355 assembled genomes were identified from isolates sequenced more than once (median (range) 2 (2–27) times). One genome was selected at random for each isolate and compared to all others from the same isolate (*n*=259 comparisons). At 95 % blast percent identity defining gene presence/absence, the median (range) gene differences between replicate sequences was 95 (28-831), showing the extent to which this metric may be affected by factors other than biological variation. Sourmash distance ranged from 0.914 to 1 with a median of 0.994. Number of genes differing between replicates was highly correlated with sourmash distance (Spearman’s rho=0.85, *P*<0.001).

### Sourmash search for *in silico* ribotype prediction

As the majority of historical surveillance data has been based on ribotypes it is natural to ask whether sourmash signatures could be used to predict ribotypes from WGS to enable continuity. We investigated whether sourmash search can be used to query a sourmash index (database) of genome signatures with known ribotypes to rapidly assign ribotypes to the search genome signature. We predicted ribotypes for 1000 randomly selected test genomes from 95 ribotypes by considering the ribotypes of the five closest matching genomes in a database (sourmash index) of 2937 genomes from 154 ribotypes. Two rules were tested, requiring 5/5 or 4/5 ribotype concordance among the signatures predicted to be most similar to the search genome signature. The five most common test ribotypes searched were RT027 (*n*=197), RT078 (*n*=90), RT014 (*n*=87), RT002 (*n*=65) and RT015 (*n*=65). There were 51 test ribotypes with <5 signatures in the database and 46 with <4; both including 18 with none.

Taking the five sequences with the closest sourmash distances per test signature, 4422/5000 (88.4 %) ribotypes matched, with estimated sourmash percent identity ranging from 77.7–100.0 % for correct ribotype matches and 52.3–100.0 % for incorrect ribotype matches. Of the 95 test ribotypes, 25 never had a correct individual match, resulting in incorrect ribotype predictions. Of these 25 ribotypes (23 of which were represented only once in the test dataset and two twice), 18 were only present in the test dataset and not in the search database.

Requiring all five database matches to be the same ribotype resulted in 780 (78 %) correctly predicted ribotypes, 200 (20 %) undeterminable (not all five top matches the same ribotype) and 20 (2 %) incorrect predictions (i.e. all five top matches were the same incorrect ribotype) (Table 3). Sensitivity excluding test genomes with ribotypes not present five times or more in the search database was 83.3 % (780/936). Relaxing the classifier to allow 4/5 concordant matches improved correctly predicted ribotypes to 872 (87.2 %) and reduced unknowns to 78 (7.8 %), but increased incorrect predictions to 50 (5 %) (Table 3). For individual matches, none correctly predicted the searched ribotype when the sourmash search identity cutoff was below 77.7 %. Applying a distance cutoff to individual matches comprising concordance or non-concordance to the 4/5 classifier decreased some incorrect calls, identifying them instead as unknowns (Table 3). Sensitivity excluding test genomes with ribotypes not present four times or more in the search database was 92.4 % (872/944). Of the 78 test ribotypes that could not be determined with this rule, 30 were due to lack of sufficient presence in the database. The 50 incorrect predictions were from 33 non-redundant searched and matched ribotype pairs, of which 26 shared an ST. For the five most common ribotypes, RT002 and RT015 were always correctly predicted (both 65/65), RT027 was almost universally correctly predicted (196/197, with one deemed undeterminable), with high rates also for RT078 (86/90; two incorrect and two undeterminable). RT014 was predicted correctly in 78/87 searches with five incorrect and four undeterminable predictions.

**Table 3.** Performance of ribotype prediction for 1000 genomes. Taking the five closest matches to a database of genome signatures (sourmash search) or full genomes (alignment) and predicting the searched genomes ribotype based on 5/5 or 4/5 concordance. Results would be reported as inconclusive when they lacked concordance, correct when the concordant ribotypes matched the searched and incorrect when they didn’t. A percent cutoff was further applied below which no individual matches had the same ribotype as the searched genome signature

Rule	Correctly determined	Incorrectly determined	Determined inconclusive
**Sourmash search closest 5/5**	780	20	200
**Sourmash search closest 4/5**	872	50	78
**Sourmash search closest 5/5 and % identity cutoff**	780	20	200
**Sourmash search closest 4/5 and % identity cutoff**	872	45	83
**SNP distance closest 5/5**	792	20	188
**SNP distance closest 4/5**	863	31	106

For comparison, we also determined ribotype predictions using core SNP distances to evaluate the potential of a distance-based approach without the noise introduced by the sourmash approach. Requiring 4/5 matching resulted in 863 (86 %) correct ribotype predictions, 106 unknown (11 %) and 31 incorrect (3 %) (Table 3). The number of correct predictions was nine fewer than from sourmash search but with 19 fewer incorrect predictions. k-folds cross-validation (k=10) was used to estimate the generalisability of this k-nearest neighbours classifier (k-NN) approach. The mean sensitivity of k-NN ribotype prediction from SNP distances was 87.2 % with a range of 82.5–89.3 % with the 4/5 rule. The mean proportion across folds of incorrect calls was 4.3 % and unknown 8.6 %. Taking reported-undeterminable that are present in <4 genomes in the index set as true negatives and those in ≥4 as false negatives, mean accuracy was calculated as 90.9 % with a range of 86.5–92.9 %.

## Discussion

The use of genomic epidemiology for pathogen surveillance depends on the ability to compare potentially large numbers of genomes that may also be diverse. Hashing k-mers and randomly subsampling is fast and scalable making it possible to compare large numbers of genomes. It has previously been shown that serovars of *Salmonella* can be differentiated with mash with high accuracy [37]. Building on these findings we investigated whether even more similar *C. difficile* genomes could be identified with k-mer hash set based approaches (full Jaccard distance) and k-mer hash subsampling (MinHash). In real genome data both full k-mer sets and k-mer subsamples comparisons were partially successful. We investigated with simulated genomes, replicate sequences and by correlation with real genome features the possible main performance factors.

Using a diverse set of *C. difficile* genomes, we demonstrate k-mer based approaches can be used to reduce the search space for subsequent comparisons by identifying potentially closely related genomes. The performance observed for highly similar genomes required using genome assembles rather than raw reads, filtering assemblies to avoid contamination, removal of excessively fragmented assemblies and removal of small contigs (<1000 bp). Removing small contigs prevented some outlying results where genomes appeared more distantly related than they were by sourmash distance. Many genome pairs with small SNP distances also reported many whole gene differences that were correlated with increased sourmash distance. Similarly, we observed up to several hundred reported gene differences in sequences generated from the same isolate or same pool of extracted DNA, raising questions as to how many reported gene differences are relevant to transmission analysis versus artefactual from the sequencing process. Simulations of genome pairs without accessory genes suggested that sequencing errors could be a major driver of poor concordance between SNP and sourmash distance. The largest available k-mer size with sourmash was selected (k=51) in order maximise discriminatory power. Large k-mers avoid the likelihood of false positive matches from too small k-mers [23]. The size of k being an odd number avoids palindromic reverse-complement sequences. Further work could involve benchmarking of optimal length k-mers against core-genome SNP distances [38, 39].

In a diverse dataset, clustering genomes by MinHash could rapidly exclude the majority of dissimilar genome pairs from further alignment and fine-scaled analysis. The genomes clustered by MinHash were comprised of more similar pairs, comparable with genomes clustered by fractional typing schemes such as ribotyping. While ribotyping is not readily possible *in silico*, seven gene MLST is, including from raw sequencing reads. MLST carries the disadvantage of incomplete schemes and un-typeable genomes but the benefit of known inconclusive results. A MinHash threshold for clustering could be suggested from our results for *C. difficile* diverse lineage exclusions, but the possibility of false negatives (even greater than observed in this study) cannot be excluded. However, MinHash clustering of similar genomes and further alignment could be conducted, rapidly correctly identify outbreaks while alignment-based investigation of the full sample of genomes is ongoing.

We also explored the possibility of a rapid distance-based ribotype prediction method. Subsampling k-mers, using sourmash search and a match consensus rule from a database (sourmash index) of genomes with known ribotypes successfully identified the correct ribotype for most searched genomes. The performance of this method was comparable to using core genome SNP distances, such that the speedup from MinHash did not introduce substantial error. With cross validation of SNP distance-based k-NN prediction, the random selection of train and test data was shown to be appropriate and robust to training input data variation. Mean accuracy was 90.6 % which approaches *in silico Salmonella* spp. serotype prediction with SISTR (94.6%) [40]. With greater numbers of ribotypes represented as well as more genomes from each ribotype it is likely that performance of this method could be improved. Direct *in silico* prediction of all known ribotypes however (as with MLST) remains an unsolved challenge, that may become more tractable as read lengths increase sufficiently to allow more complete genome reconstruction. Where other traits are associated with overall population structure it is also possible to use k-mer based approaches for predicting these, e.g. of antimicrobial resistance [41].

In conclusion, k-mer based approaches to comparing *C. difficile* assembled genomes at scale offered modest performance. In a genomic surveillance context where hundreds or thousands of genomes for comparison are becoming routine, it does provide the opportunity to computationally inexpensively and rapidly subset genomes for alignment and outbreak detection while full, fine-scaled investigations are ongoing.

## Supplementary Data

Supplementary material 1

Supplementary material 2

Supplementary material 3

Supplementary material 4

Supplementary material 5

Supplementary material 6

Supplementary material 7
